# Accuracy of time to treatment estimates in the CRASH-3 clinical trial: impact on the trial results

**DOI:** 10.1186/s13063-020-04623-5

**Published:** 2020-07-25

**Authors:** Raoul Mansukhani, Lauren Frimley, Haleema Shakur-Still, Linda Sharples, Ian Roberts

**Affiliations:** 1 Clinical Trials Unit, London School of Hygiene & Tropical Medicine, Keppel Street, London, WC1E 7HT UK; 2 Department of Medical Statistics, London School of Hygiene & Tropical Medicine, Keppel Street, London, WC1E 7HT UK

**Keywords:** Antifibrinolytic, Tranexamic acid, measurement error, Traumatic brain injury, Monitoring

## Abstract

**Background:**

Early treatment with tranexamic acid may reduce deaths after traumatic brain injury (TBI). In mild and moderate TBI, there is a time to treatment interaction, with early treatment being most beneficial. Time to treatment was recorded by clinicians and is subject to error. Using monitoring data from the CRASH-3 trial, we examine the impact of errors in time to treatment on estimated treatment effects.

**Methods:**

The CRASH-3 trial was a randomised trial of the effect of tranexamic acid on death and vascular occlusive events in 12,737 TBI patients. This analysis includes the 8107 patients with a Glasgow coma scale score of 9 to 15 since previous analyses showed that these patients benefit most from early treatment. Clinician-recorded time to treatment was checked against ambulance and hospital records for 1368/12,737 (11%) patients. Patients who died were preferentially selected for monitoring and we monitored 36% of head injury deaths. We describe measurement errors using Bland-Altman graphs. We model the effect of tranexamic acid on head injury death using logistic regression with a time-treatment interaction term. We use regression calibration, multiple imputation and Bayesian analysis to estimate the impact of time to treatment errors.

**Results:**

Clinicians rounded times to the nearest half or full hour in 66% of cases. Monitored times were also rounded and were identical to clinician times in 63% of patients. Times were underestimated by an average of 9 min (95% CI − 85, 66). There was more variability between clinician-recorded and monitored times in low- and middle-income countries than in high-income countries. The treatment effect estimate at 1 h was greater for monitored times OR = 0.61 (95% CI 0.47, 0.81) than for clinician-recorded times OR = 0.63 (95% CI 0.48, 0.83). All three adjustment methods gave similar time to treatment interactions. For Bayesian methods, the treatment effect at 1 h was OR = 0.58 (95% CI 0.43, 0.78). Using monitored times increased the time-treatment interaction term from 1.15 (95% CI 1.03, 1.27) to 1.16 (95% CI 1.05, 1.28).

**Conclusions:**

Accurate estimation of time from injury to treatment is challenging, particularly in low resource settings. Adjustment for known errors in time to treatment had minimal impact on the trial results.

**Trial registration:**

ClinicalTrials.gov NCT01402882. Registered on 25 July 2011

## Introduction

In emergency situations, treatment effects may depend on the time delay between the acute event and administration of the trial intervention. The CRASH-3 trial [1] showed that in patients with mild and moderate head injuries, tranexamic acid treatment reduced head injury deaths. As in previous trials of tranexamic acid in life-threatening bleeding, there was a strong time to treatment (TTT) interaction. The CRASH-2 trial [2] examined the effects of tranexamic acid in bleeding trauma patients. The WOMAN trial [3] examined its effects in post-partum haemorrhage. Both trials showed that tranexamic acid reduces death from bleeding when given within 3 h of bleeding onset with no benefit when given after 3 h. An individual patient data meta-analysis [4] found that for every 15-min treatment delay, there was a 10% reduction in effectiveness.

The CRASH-3 trial was conducted in 175 hospitals in 29 countries. Many patients were recruited in countries without formal pre-hospital emergency medical services (e.g. ambulance systems) and patients were often taken to hospital by bystanders or family members in taxis or private vehicles. In these cases, the time of injury was not formally recorded and was estimated by a clinician based on the location of injury and approximate transport times. However, in low- and middle-income countries, patients are often taken to the nearest primary healthcare centre, where they receive basic first aid before transfer to a tertiary hospital. In these situations, estimating the time of injury using location of injury and transportation times can be highly inaccurate.

Random measurement error can bias estimates of regression coefficients, reducing the apparent association between an exposure and outcome [5, 6]. Error in clinician-recorded TTT could obscure or weaken the TTT interaction and this could have clinical implications. In clinical trials, risk-adapted approaches to monitoring include verifying a proportion of participants’ measurements to assess the extent and nature of any errors and to adjust the analysis if necessary [7]. We examined the impact of mismeasurement in clinician-recorded TTT on treatment effects. We used three established statistical methods to correct for mismeasurement in clinician-recorded TTT using a sample of monitored patients.

## Methods

We examined data from the CRASH-3 trial, a randomised trial of the effect of tranexamic acid on death, disability and vascular occlusive events in 12,737 TBI patients. The inclusion criteria were: adults with TBI, who had a Glasgow coma scale score (GCS) ≤ 12 or any intracranial bleeding on CT scan and no significant extra-cranial bleeding. The primary outcome was head injury death within 28 days. Of the 12,737 patients randomised, clinician-recorded TTT was monitored for 1368 (11%) patients by comparing clinician-recorded times with those based on data from ambulance and hospital records.

In this analysis, we examine the effect of inaccuracy in TTT estimates in 8107 patients with mild and moderate head injury. In this population, there was evidence of benefit from tranexamic acid treatment and evidence of a time treatment interaction. Patients with mild to moderate TBI had a baseline GCS of 9 to 15, and of these, 456 (6%) were monitored. Patients who died were preferentially chosen for monitoring. Hospitals that recruited a larger number of patients were monitored by visit. Other hospitals were monitored by telephone. All patient details (including TTT, GCS, age and SBP) were checked.

We estimated odds ratios and 95% confidence intervals for the effect of tranexamic acid on head injury deaths according to TTT using a multivariable logistic regression model (1):

1 $$ \mathrm{logit}\left(p\left(Y=1\right)\right)={\beta}_0+{\beta}_1X+{\beta}_2T+{\beta}_3X\ast T+{\beta}_4 GCS+{\beta}_5 SBP+{\beta}_6A $$

(*Y* = 1 is head injury death within 28 days, *X* is treatment (tranexamic acid = 1, placebo = 0), *T* is TTT in hours, *A* is age in years at the time of the acute event, *GCS* is Glasgow coma scale and *SBP* is systolic blood pressure in mmHg). We assume that clinician-recorded time is measured with error and that the monitored time more accurately reflects TTT.

Three methods (regression calibration, multiple imputation and a full Bayesian analysis) were used to adjust for mismeasurement in clinician-recorded time. Each required the following two steps,

1. A model for the association between clinician-recorded and monitored time was estimated from the sample of patients who were monitored and
2. This fitted model was used to impute a monitored time for the patients who were not in the monitoring sample.

Within this framework, monitored time was assumed to have a linear relationship with clinician time.

2 $$ {T}_M={\alpha}_0+{\alpha}_1{T}_C+{\alpha}_2A+{\alpha}_3 SBP+{\alpha}_4 GCS+e $$

where *e*~*N*(0, *σ*^2^), *T*_*M*_ is the monitored time and *T*_*C*_ is the clinician-recorded time.

First, for regression calibration, model coefficients (Eq. 2) were estimated using the monitored data and used to predict times in the unmonitored population. Confidence intervals were calculated by bootstrapping. Second, we treated unmonitored patients as having missing data and used multiple imputation as described by Bartlett [8, 9]. Third, we examined the impact of measurement error in a full Bayesian model, in which monitored TTT for patients who are not monitored was treated as another parameter to be estimated. For all three methods, the effect of tranexamic acid on death within 28 days was estimated from the model in Eq. 1 based on the actual data for monitored patients and imputed values for unmonitored patients. Further details of the methods are given in the statistical methods section of the Additional file 1.

## Results

Of the 8107 patients with mild and moderate head injury, 537 (7%) died from head injury within 28 days. Of the 456 monitored patients, 186 (41%) died from head injury within 28 days. Table 1 shows the characteristics of the included patients stratified by monitoring status. In 63% (287/456) of monitored patients, clinician-recorded times and monitored time were the same. Clinician-recorded times were less than monitored times for 28% (128/456) and more than monitored times for 9% (41/456) of monitored patients.

**Table 1:** The characteristics of the included patients stratified by monitoring status

	Monitored	Unmonitored
	(*n* = 456)	(*n* = 7651)
**Received TXA**	229 (50%)	3852 (50%)
**Head injury death**	186 (41%)	351 (5%)
**Time to treatment**		
Mean (SD)	156 min (86 min)	178 min (108 min)
≤ 1	51 (11%)	955 (12%)
1–2	128 (28%)	2242 (29%)
2–3	185 (41%)	2117 (28%)
3–4	36 (8%)	705 (9%)
4–5	18 (4%)	572 (7%)
5–6	21 (5%)	500 (7%)
6–7	10 (2%)	375 (5%)
7–8	7 (2%)	185 (2%)
**Sex**		
Male	368 (81%)	5937 (78%)
Female	88 (19%)	1713 (22%)
Unknown	0 (0%)	1 (0%)
**GCS**		
Moderate 9–12	297 (65%)	3535 (46%)
Mild 13–15	159 (35%)	4051 (53%)
Unknown	0 (0%)	65 (1%)
**SBP (mmHg)**		
0–89	7 (2%)	57 (1%)
90–119	94 (21%)	2546 (33%)
120–139	166 (36%)	2730 (36%)
140+	189 (41%)	2300 (30%)
Unknown	0 (0%)	18 (0%)
**Age (years)**		
Mean (SD)	51 (22)	43 (20)
0–15	0 (0%)	2 (0%)
16–24	62 (14%)	1628 (21%)
25–34	79 (17%)	1566 (20%)
35–44	42 (9%)	1172 (15%)
45–54	67 (15%)	1082 (14%)
55+	206 (45%)	2201 (29%)

Baseline characteristics of mild and moderately injured patients in CRASH-3 by monitoring status

*N* = 8107

**Fig. 1:**
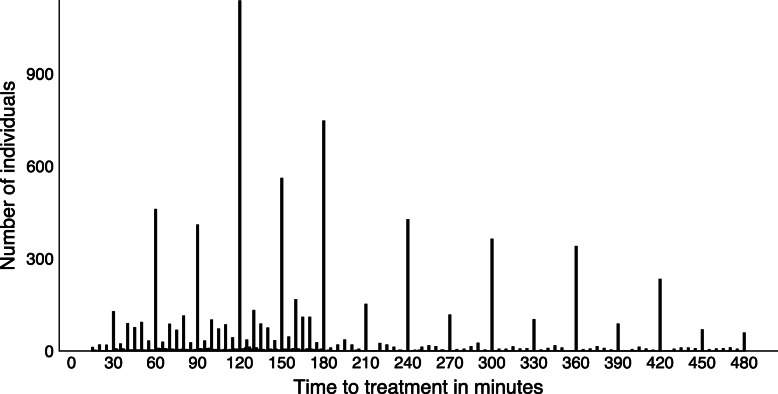
Histogram showing digit preference in time to treatment for mild to moderately injured patients in CRASH-3. *N* = 8107. Time is from monitoring where available else clinician-recorded

Figure 1 shows a histogram of clinician-recorded times. The most common TTT is at 2 h with the next most commonly occurring TTT’s being at 3 h, 2 h 30 min, 1 h and 4 h. There was strong digit preference with times rounded to half an hour in 20% and to the hour in 46% of patients. There was also strong digit preference in the monitored times with times rounded to half an hour in 21% and to the hour in 29% of patients (Additional file 1: Figure 1).

**Fig. 2:**
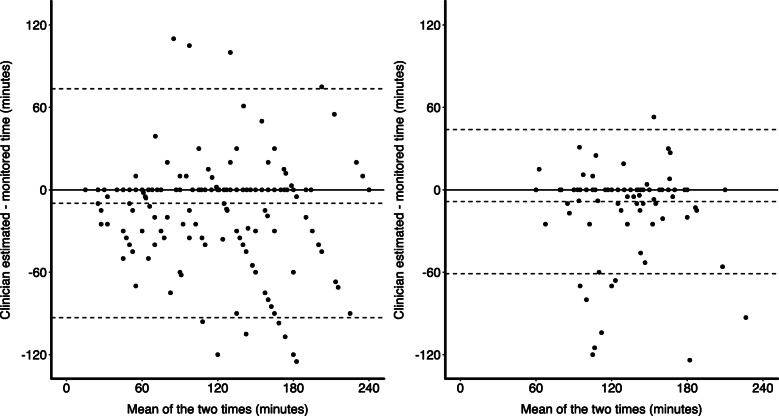
Bland-Atman graphs by country income level. The graph on the left is for low- and middle-income countries (*N* = 319, bias = − 10 min, upper limit of agreement = 74 min, lower limit of agreement = − 93 min). The graph on the right is for high-income countries (*N* = 137, bias = − 9 min, upper limit of agreement = 44 min, lower limit of agreement = − 61 min). The magnitude of the bias and the gap between the limits of agreement are larger in low- to middle-income compared to high-income countries

The mean difference between clinician-recorded and monitored TTT was − 9 min, indicating that clinicians were more likely to underestimate TTT on average. The differences ranged from the clinician-recorded time being 66 min larger to 85 min smaller than the monitored time (Additional file 1: Figure 2). Figure 2 shows Bland-Altman graphs of clinician-recorded versus monitored TTT by country income level. In low- and middle-income countries, the mean difference was − 10 min. The differences ranged from the clinician-recorded time being 74 min larger to 93 min smaller than the monitored time. In high-income countries, the mean difference was − 9 min. The differences ranged from the clinician-recorded time being 44 min larger to 61 min smaller than the monitored time. The standard deviation of the time difference was 38 min for all countries combined, 42 min in low- and middle-income countries and 27 min in high-income countries. There was strong evidence that this time difference variance was larger in low- and-middle compared to high-income countries (*F*_318,136_ = 2.52, *p* < 0.0001).

Using regression and assuming a linear relationship between monitored and clinician time (Eq. 2) *R*^2^ = 0.82.

Table 2 and Fig. 4 show the ORs and 95% CIs for the effect of tranexamic acid by TTT based on clinician-recorded time, monitored time and after using the three statistical adjustment methods. The interaction term is larger when monitored times were used. The adjustment methods all give similar results in the CRASH-3 data and increase the time treatment interaction further.

**Table 2:** The ORs and 95% CIs for the effect of tranexamic acid by TTT based on clinician-recorded time, monitored time and after using the three statistical adjustment methods

	Clinician est. time	CRASH-3 monitored time	Regression calibration	Multiple imputation	Bayesian methods
**Interaction**	1.15 (1.03, 1.27)	1.16 (1.05, 1.28)	1.19 (1.05, 1.34)	1.18 (1.05, 1.33)	1.18 (1.06, 1.34)
**0 h**	0.55 (0.38, 0.78)	0.53 (0.37, 0.76)	0.49 (0.32, 0.73)	0.50 (0.33, 0.75)	0.49 (0.33, 0.73)
**1 h**	0.63 (0.48, 0.83)	0.61 (0.47, 0.81)	0.58 (0.42, 0.79)	0.59 (0.43, 0.80)	0.58 (0.43, 0.78)
**2 h**	0.72 (0.58, 0.89)	0.71 (0.58, 0.88)	0.69 (0.55, 0.86)	0.69 (0.56, 0.87)	0.69 (0.55, 0.86)
**3 h**	0.83 (0.69, 1.00)	0.83 (0.69, 0.99)	0.82 (0.68, 0.99)	0.82 (0.68, 0.99)	0.82 (0.68, 0.98)
**4 h**	0.95 (0.76, 1.18)	0.96 (0.77, 1.19)	0.97 (0.78, 1.22)	0.97 (0.78, 1.21)	0.97 (0.78, 1.21)
**5 h**	1.09 (0.82, 1.44)	1.11 (0.84, 1.47)	1.15 (0.85, 1.58)	1.15 (0.84, 1.56)	1.15 (0.85, 1.55)
**6 h**	1.25 (0.87, 1.80)	1.29 (0.89, 1.85)	1.37 (0.91, 2.10)	1.35 (0.90, 2.03)	1.36 (0.92, 2.03)
**7 h**	1.43 (0.91, 2.27)	1.49 (0.94, 2.35)	1.63 (0.97, 2.80)	1.60 (0.95, 2.68)	1.61 (0.98, 2.68)
**8 h**	1.64 (0.94, 2.86)	1.73 (0.99, 3.00)	1.94 (1.02, 3.75)	1.89 (1.00, 3.55)	1.90 (1.04, 3.55)

The effect of monitoring and statistical adjustment methods on the results of CRASH-3 for mild and moderately injured patient. *N* = 8107. There are 537 head injury deaths in this population. Monitoring of TTT was carried out on 456 individuals. Interaction refers to the time treatment interaction term in the substantive model (Eq. 1). The “0 h,” “1 h,” etc., row headings refer to the treatment effect OR at that time point

**Fig. 3:**
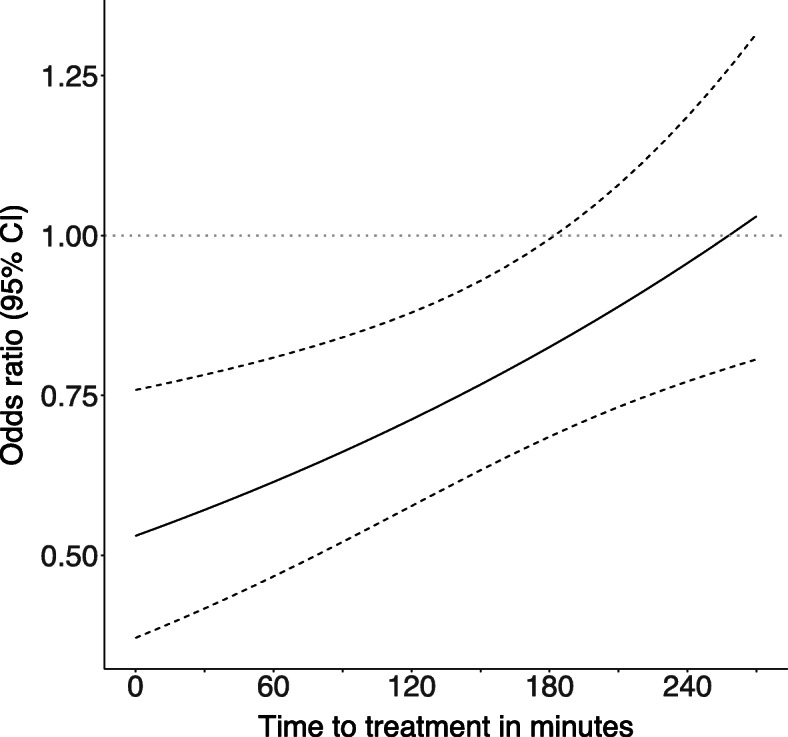
Tranexamic acid effectiveness in preventing death due to TBI versus time to treatment. Mild and moderately injured patients only. *N* = 8107, number of head injury deaths = 537. Time to treatment is from monitoring where available else clinician-recorded

Figure 3 shows the effect of tranexamic acid on head injury death by TTT (and provides a graphical representation of the first column of Table 2). The odds ratio for the treatment effect increases with time. There is a 10% reduction in treatment effectiveness for every 20-min increase in TTT (Fig. 3).

**Fig. 4:**
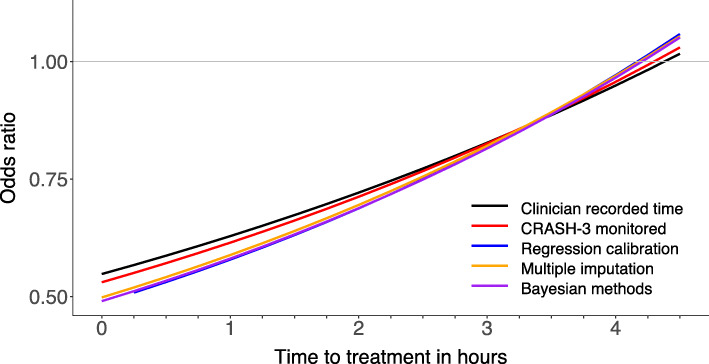
The effect of monitoring and statistical adjustment methods on the treatment effectiveness of tranexamic acid verses time to treatment. *N* = 8107, number of head injury deaths = 537. CRASH-3 monitored time consists of monitored time where available else clinician-recorded

## Discussion

In the CRASH-3 trial, clinicians often underestimated TTT with mismeasurement being greater in low- and middle-income countries than in high-income countries. Adjustment for known errors in TTT had little impact on the results although effect estimates were slightly larger. Our results suggest that early treatment with tranexamic acid might be even more important than previously reported.

We restricted our analysis to mild and moderately injured patients since there was a strong time treatment interaction in this subgroup. We excluded severely injured patients because there was no evidence of a time treatment interaction in these patients [1].

In over half of monitored patients, clinician-recorded and monitored times were identical. However, this does not mean that clinician-recorded times are accurate. For many patients, monitors had no additional information on which to base their assessments and so it is not surprising that the clinician-recorded times did not change after monitoring. There was strong digit preference in both clinician-recorded and monitored times which suggest inaccuracy in both sets of measurements. Time of treatment is usually recorded in the patient notes but the time of injury is often uncertain. In high-income countries, ambulance records often provide information on the approximate time of injury, but in low- and middle-income settings, these records are often absent. Accordingly, the variance of the difference between clinician-recorded and monitored TTT was larger in low- and middle-income compared to high-income countries. In low- and middle-income settings, time of injury was often estimated from bystander reports of the location of injury and estimated travel times to the treating hospital. However, because patients are often taken to the nearest healthcare centre before transfer to the randomising hospital, this method can lead to substantial underestimation of the time since injury.

It seems reasonable to assume that clinicians are more likely to underestimate than overestimate TTT. This assumption is consistent with the general psychological literature of time perception and with studies of time to treatment estimation in trauma patients [10–14]. In this study, clinician-recorded TTT was less than monitored TTT for 28% and more than monitored TTT for 9% of monitored individuals. It is well known that random mismeasurement of an exposure variable biases its apparent effect on the outcome variable towards zero [5, 6]. In the CRASH-3 trial, there was a small increase in treatment effect after adjustment for mismeasurement. Because we could not fully adjust for mismeasurement, it is likely that the treatment effect and particularly its interaction with TTT may be underestimated.

Accurate estimation of time to treatment is also important in stroke [15] and myocardial infarction [16] where treatment is only effective if given within a limited time window. In both of these areas, novel alternatives to patient reported times have been proposed [17, 18].

The three statistical methods, each of which assume a linear relationship between monitored and clinician time, gave similar results. Bartlett [19] found that the full Bayesian analysis gave more biased results than regression calibration for small effect sizes when the reliability of the imputation model was low. However, regression calibration can underestimate regression coefficients for large effect estimates [20]. Neither scenario is true for these data. Having 94% missingness is unusual when imputing missing data. However, in this case, we have a large number (456) of monitored times for reliable imputation. The large sample size and the large number of monitored values are important strengths of our study.

Of the three statistical adjustment methods, we found regression calibration the easiest to implement requiring only a small amount of non-standard code to estimate confidence intervals by bootstrapping. For MI and the full Bayesian analysis, a number of freely available software packages are available (for example JAGS [21], OpenBUGS [22], STAN [23], SMCFCS [24]). However, these packages are not routinely used by applied statisticians in trials units.

## Conclusions

Randomised trials of potentially time critical treatments need to consider measurement error in estimated TTT. Validation studies may be necessary but identifying the gold standard measurement is challenging in acute settings.

## Supplementary information

**Additional file 1.**

## Data Availability

Following publication of the primary and secondary analyses detailed in the statistical analysis plan [25], individual de-identified patient data, including a data dictionary, will be made available via our data sharing portal, The Free Bank of Injury and Emergency Research Data (freeBIRD) website at https://ctu-app.lshtm.ac.uk/freebird/.

## Abbreviations

CRASH-2: Clinical Randomisation of an Antifibrinolytic in Significant Haemorrhage
CRASH-3: Clinical Randomisation of an Antifibrinolytic in Significant Head injury
GCS: Glasgow coma scale score
MI: Multiple imputation
OR: Odds ratio
SBP: Systolic blood pressure in mmHg
TBI: Traumatic brain injury
TTT: Time to treatment
WOMAN: World Maternal Antifibrinolytic
