# Supplementary material for: Accuracy of time to treatment estimates in the CRASH-3 clinical trial: impact on the trial results
Source: Trials. 2020 Jul 25;21:681. doi: 10.1186/s13063-020-04623-5 (PMC7382791; doi:10.1186/s13063-020-04623-5)
Supplement: Supplementary file 1 — Additional file 1. [file 13063_2020_4623_MOESM1_ESM.docx]

# Appendix

## Statistical Methods

**Methods A1: Brief description of the Multiple imputation process.**

The MI process involves estimating the imputation model alpha coefficients and their variance covariance matrix via maximum likelihood. M random draws of the alpha coefficients are made from the estimated joint distribution of the alpha coefficients. M imputed datasets are created by estimating monitored time based on the imputation model and the M random draws of the alpha coefficients. The substantive model is fitted using each of the M complete datasets. The beta coefficients for the M imputed datasets are then combined using Rubin’s rules to provide a single multiple imputation result set.

We used the SMCFCS package^1^ with-in R for our MI analysis.

**Methods A2: Brief description of how we implemented Full Bayesian Analysis**

Marginal Posterior distributions of the alpha and beta parameters for equation (1) and (2) were obtained using Markov Chain Monte Carlo (MCMC). Mildly informative priors of N(0,100) were used for the alpha and beta parameters and a mildly informative prior of Uniform(0,2.5) was used for the variance of the error term in equation (2). We used the Just Another Gibbs Sampler^2^ (JAGS) software package with-in R for our analysis. The point estimates of interest were calculated from the mean of the posterior distribution of the beta coefficients. Our credible interval range was the 2.5% and 97.5% centile of the posterior distribution of the beta coefficients. Three independent chains were used. Chain convergence was checked for by visual inspection of the history and Brooks-Gelman-Rubin diagnostic plots. Keogh^3^ and Bartlett^4^ describe in detail how to use a fully Bayesian analysis in a measurement error scenario.

**Methods A3: Effectiveness calculation**

The calculation for the effectiveness at tranexamic acid at a given time t is based on the odds ratio (OR) from the substantive model [1]. We used monitored time where available else clinician-recorded time. We calculate OR­_2_=1/OR the odds ratio for not dying due to head injury at a given time t in the tranexamic acid versus placebo arm. The effectiveness of tranexamic acid at time t is then calculated as [(**OR_2_ at time t)**-1]/ [(**OR_2_ at time zero)** - 1]

Using the data from CRASH-3 for mild and moderately injured patients:

We calculate from the substantive model (1) the OR­_time=0_ =0.530 and OR_time=20minutes_=0.557

Effectiveness = 100* [(**OR_2_ at time t)**-1]/ [(**OR_2_ at time zero)** - 1] where OR_2_=1/OR

Effectiveness % at 20 minutes = 100* [(1/0.557)-1]/[(1/0.530)-1]=0.897 ≈ 10 % reduction in effectiveness for every 20 minute delay.

Figures****

**Figure A1: Histogram showing digit preference in monitored times to treatment for mild and moderate (GCS≥9) patients in the CRASH-3 trial. N=456**

**Figure A2: Bland Altman graph of clinician recorded and monitored times in mild to moderately injured patients in CRASH-3. N=456, bias = -9 minutes, upper limit of agreement = 66 minutes, lower limit of agreement = -85 minutes.**

## References

| 1 | Bartlett JW, Keogh RH. SMCFCS: Multiple imputation of covariates by substantive model compatible fully conditional specification, 2019.  <https://cran.r-project.org/package=smcfcs> Accessed 14 Feb 2020 |
| --- | --- |
| 2 | Plummer M (2003). JAGS: A Program for Analysis of Bayesian Graphical Models Using Gibbs Sampling, Proceedings of the 3rd International Workshop on Distributed Statistical Computing (DSC 2003), March 20–22, Vienna, Austria. ISSN 1609-395X |
| 3 | Keogh RH, Bartlett JW. Measurement error as a missing data problem. 2019; Available from: <http://arxiv.org/abs/1910.06443> |
| 4 | Bartlett JW, Keogh RH. Bayesian correction for covariate measurement error: A frequentist evaluation and comparison with regression calibration. Stat Methods Med Res [Internet]. 2018 Jun 28;27(6):1695–708. Available from: <https://doi.org/10.1177/0962280216667764> |
|  |  |
|  |  |
|  |  |
